# Bioboxes: standardised containers for interchangeable bioinformatics software

**DOI:** 10.1186/s13742-015-0087-0

**Published:** 2015-10-15

**Authors:** Peter Belmann, Johannes Dröge, Andreas Bremges, Alice C. McHardy, Alexander Sczyrba, Michael D. Barton

**Affiliations:** 1 Faculty of Technology and Center for Biotechnology, Bielefeld University, 33615 Bielefeld, Germany; 2 Computational Biology of Infection Research, Helmholtz Centre for Infection Research, 38124 Braunschweig, Germany; 3 DOE Joint Genome Institute, Walnut Creek, CA 94598 USA

**Keywords:** Bioinformatics, Software, Docker, Standards, Usability, Reproducibility

## Abstract

Software is now both central and essential to modern biology, yet lack of availability, difficult installations, and complex user interfaces make software hard to obtain and use. Containerisation, as exemplified by the Docker platform, has the potential to solve the problems associated with sharing software. We propose bioboxes: containers with standardised interfaces to make bioinformatics software interchangeable.

The increasing size of datasets in biology has demanded a corresponding increase in the reliance on software to automate tasks that have become impossible to do manually [1]. Improvements in technology have made creating large biological datasets much easier, so that small facilities can now feasibly do large-scale genomic sequencing or proteomics. Biology in 2015 may require a researcher to be using a pipette one day and running genome assembly software the next. As the field changes to where scientists are expected to regularly use bioinformatics tools, the difficulties in sharing academic software are becoming a hindrance to both research and publication.

Bioinformatics software is seen as research output and published as journal articles. It then follows that a bioinformatics software developer is measured by the same metric as is often used for a biologist studying novel protein mechanisms - the more articles the better. This has lead to proliferation of bioinformatics software; for example, the Wikipedia page for sequence aligners alone lists 64 different implementations.

Perverse incentives have lead to a ‘fire-and-forget’ approach to software; publishing multiple software articles is rewarded whereas there are no direct metrics for maintaining existing software [2]. The status quo serves authors by allowing them to generate long publication lists, and serves publishers by generating revenue through article processing fees. The intended audience, the biologists trying to do research, are then left to wade through a corpus of buggy, inconsistent, and confusing tools [3]. This commentary addresses three prominent symptoms of this system: lack of software availability, difficulty installing software, and divergent formats and interfaces for common tasks.

## Lack of software availability

A ridiculous situation in the publication system is that publishing an article about software does not guarantee that the tool is actually available for use. Unless the journal specifically mandates the use of a third party service to host the software, the article may describe the tool as being "available on request" - the reader has to contact the author for access. If the author cannot (or does not want to) be contacted, the software essentially ceases to exist.

Another common situation is that software is unavailable because the developer has moved on to a different position, or because the research funding supporting their position ends. This results in the developer no longer being able to maintain the software, the website describing the tool no longer existing, or both. A study related to this effect showed that, in many cases, bioinformatics web addresses are often not available 2-3 years after the article describing it was published [4].

## Difficulty installing software

Differences between operating systems and hardware require effort in delivering software in a ready-to-use form. One example is when software is written in a language such as C/C++ that must be 'compiled' to generate platform-specific executable files. Compiling, however, is not a simple task, and it is even harder in biology because of the limited experience that biologists may be expected to have with C++ build tools. Alternatively, even if tools are written using 'platform-independent' languages such as Java or Python, these often have third-party dependencies that must also be downloaded and installed, requiring additional time and effort.

As software delivery and maintenance plays no part in the publication process, the biologist is usually left with the work of compiling source code, manually installing required software libraries and debugging platform-incompatible code: for instance, having to decipher obscure output such as the GNU make command reporting a g++ error because the wrong version of libboost-dev is installed.

## Lack of standards

Every piece of bioinformatics software has a different interface even when performing the same kind of operation. No two short read aligners may be expected to use the same method to identify the input file for processing in the command line options; for example, this might be --input, --fastq or the position in the input arguments. The output BAM file, a standard format for sequence alignment, generated by a short read aligner may be created in different locations, or the output may not be stored in BAM at all. Across all available short read aligners this leads to a multitude of different ways of doing the same task each time: take a list of sequence reads and return a description of how they map to a reference genome.

This leads to the current situation, where researchers spend much of their time shifting data between the incompatible interfaces of different tools and converting to required data formats. Is there a good reason why short read aligners, which all do the same task, should not be standardised with the same interface? How about genome assemblers, FASTQ preprocessors or multiple sequence aligners? The tools for each of these tasks essentially perform the same operation, but each has a different interface. Again, the current situation does not serve intended users that the software is written for: biologists and bioinformaticians.

## Software containerisation and standardisation

The Docker platform [5] allows the creation of lightweight containers in which developers can install their software along with all required libraries and scripts. These containers can then be easily shared through a central repository, or as compressed files, and used in the same way as if the software itself were installed. The bioinformatics field has quickly recognised the opportunity provided by Docker [6, 7], in which containers do not dictate a specific software framework or language for implementing bioinformatics tools, and which allows integration with existing software.

Containerisation further has the potential to solve the problems of software availability and installation outlined above, where bundling all dependencies removes the need for the user to compile and install anything (except Docker itself). Software containers also provide researchers with the option to reproduce existing published results so as to replicate or expand on the work of others. An example of this are nucleotid.es [8] and the Critical Assessment of Metagenomic Interpretation (CAMI) [9] projects, where the tools benchmarked are containerised and available for download by users.

Even with these outlined advantages, without standardisation bioinformatics will continue to suffer from mismatching interfaces between tools in software pipelines. The time-consuming job of maintaining these pipelines then falls to the bioinformatician, reducing their role from computational researchers to the custodians of gluing different tools together.

To this end we, developers involved in both CAMI and nucleotid.es, have created the bioboxes project [10] with the aim of specifying standardised bioinformatics containers. A biobox is a software container with a standardised interface that describes what kind of input files and parameters are accepted and which output files are to be returned. An example is a short-read assembler that takes an input paired-FASTQ file and returns a contig FASTA file. Each developer creating a biobox should make sure the container accepts these inputs and returns the expected outputs.

**Fig. 1:**
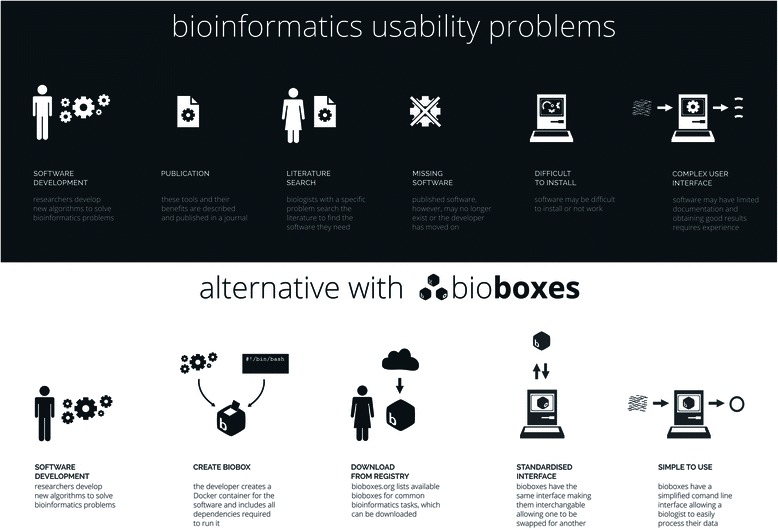
Comparison of the current software situation in bioinformatics (*top*) with using biobox Docker containers with standardised interfaces (*bottom*)

Specifying the same interface for the same task allows one tool to be swapped for another in a pipeline. This creates an interchangeable parts list for researchers, which, combined with Docker containerisation, means that biologists and bioinformaticians have access to, and can immediately use, a large body of bioinformatics software. Figure 1 contrasts the existing state of bioinformatics software with bioboxes standardised software containers. Box 1 shows a python command line interface to bioboxes that allows the reader to test out a biobox.

## Box 1: Example of bioboxes command line interface

This example requires that both python’s pip and Docker are installed. General instructions for installing pip are widely available online. Instructions for installing Docker can be found on the Docker website [5].

pip install ––user biobox_cli

biobox run short_read_assembler bioboxes/velvet ––input reads.fq.gz ––output contigs.fa

biobox run short_read_assembler bioboxes/megahit ––input reads.fq.gz ––output contigs.fa

We ask that developers try to follow the documentation on the bioboxes website [10] and contribute biobox-compatible Docker images of their software. Then, biologists and bioinformaticians, editors, and reviewers should begin requesting that biobox versions of software are available alongside publications. At the same time, the bioboxes project welcomes feedback from the community. The website [10] contains links on how to get involved by filing bug reports or asking questions in the chat room.

## Conclusions

The current state of bioinformatics software works against users by consuming time and effort, and against reproducibility by the lack of methods to recreate the work of others. The use of software containers with standardised interfaces has the potential make the work of biologists easier by creating simple to use, interchangeable tools. At the same time developers can make their programs more easily available and usable by a wider audience. Ultimately this drive for standardisation and the ensuing benefits will be successful only if they are both accepted and driven by the bioinformatics community itself.
